# Surface Electromyography Analysis of the Lower Extremities of Subjects Participating in Baduanjin Exercises

**DOI:** 10.1155/2017/1304190

**Published:** 2017-12-18

**Authors:** Li Jin, Ran Li, Jing Chen, Qinbo Xue, Yueqin Yang

**Affiliations:** ^1^College of Health Science, Wuhan Sports University, Wuhan 430079, China; ^2^Hubei Exercise Training and Monitoring Key Laboratory, Wuhan Sports University, Wuhan 430079, China; ^3^China Institute of Sport Science, General Administration of Sport of China, Beijing 100061, China

## Abstract

**Purpose:**

The purpose of this study was to assess the effects of practicing Baduanjin exercises on the lower extremities of subjects using electromyography analysis.

**Subjects:**

110 healthy adults were randomly assigned as subjects to two groups: SG group who received sixteen weeks of Baduanjin training and CG group who received no training.

**Methods:**

The methods used in this study included the use of a sixteen-channel sEMG system to record and measure activity changes in vastus medialis and vastus lateralis.

**Results:**

After 16 weeks of Baduanjin training, the results of this study showed that the SG group had significant increases in RMS (root mean square) (in vastus lateralis, *p* > 0.05; in vastus medialis, *p* < 0.05), in AEMG (average electromyographic activity) (in vastus lateralis, *p* > 0.05; in vastus medialis, *p* < 0.05), and in IEMG (integrated electromyogram) (in vastus lateralis, *p* > 0.05; in vastus medialis, *p* < 0.05). No adverse events from treatment were reported during the whole period of this study.

**Conclusion:**

This study concludes that performing 16 weeks of Baduanjin training can significantly improve strength and the physical function of the lower extremities among healthy adults.

## 1. Introduction

Baduanjin (also known as Eight Pieces of Brocade) is one of the most popular traditional exercises in China. The main forms of Baduanjin are eight separate exercises, with each exercise focusing on a different physical area and Qi meridian [1], which moves and activates all parts of the body [2]. Baduanjin is considered to be a low-intensity, aerobic exercise and is easy to learn [3], because it is less demanding of physical and cognitive skills [3, 4]. Consequently, Baduanjin is widely practiced as a safe and popular, health-promoting exercise by middle aged people in China [5].

Baduanjin is commonly compared with other forms of Qigong exercises (e.g., Tai Chi Chuan, Medical Qigong, and Yoga) and is considered generally equivalent as an exercise for the promotion of health and wellness. Research literature indicates that significant benefits in promoting health may be accrued. It is reported that regular practice of Baduanjin, as well as other fitness Qigong exercises, can have positive effects on hypertension, cardiovascular disease, cancer, arthritic disease, stroke rehabilitation, aerobic capacity, and bone mineral density [6]. Furthermore, safety and positive effectiveness in health promoting have been reported for the patients with neurological diseases, rheumatological diseases, orthopedic diseases, cardiovascular diseases, chronic obstructive pulmonary diseases, and breast cancers [7]. It is also reported that regular participation in Tai Chi Chuan can improve balance control [8], and other benefits are proved including enhancements of the knee extensor and flexor strength, proprioception, and the reflex reaction time of the lower extremities [9].

Surface electromyography (sEMG) is a noninvasive and painless way to evaluate muscle function and efficiency. And its use for detecting and recording electrical potential ensures that the results of the assessment are objective and reliable [10]. Noninvasiveness is one of the most important advantages of sEMG [11], as well as the fact that sEMG electrodes are fairly inexpensive and can be easily placed on various muscles in the body, which makes sEMG suitable for a variety of study and research purposes [12].

Both Tai Chi Chuan and Baduanjin are widely regarded as health-promoting exercises. They are classified as fitness Qigong due to their similar, meditative breath-related movement and share common Tai Chi philosophical roots, as well as common aims of promoting wellness and health [6]. Most previous studies were focused on specific diseases or symptoms, with the type of intervention selected being mainly Tai Chi Chuan (as previously noted). The value of the Baduanjin exercises for promoting health has not yet been proven. Therefore, the main purpose of this study is to assess the effects of practicing Baduanjin on the lower extremities of our subjects, using surface electromyography. It is hoped that this study will show that Baduanjin exercises do favorably promote health and improve the functioning of the lower extremities of our participants.

## 2. Materials and Methods

### 2.1. Participants

The Wuhan Sports University conducted this research project, in accordance with the ethical code of the World Medical Association. Approval had also been obtained from the Ethics Committee of the Wuhan Sports University. Recruitment of local male and female subjects took place from October 2012 to February 2013. The subjects ranged in age from 20 to 59 years and had engaged in no regular, moderate intensity physical exercise. All were physically healthy, with no cardiovascular diseases, diabetes (or abnormal glucose tolerance), or any other acute or chronic diseases that could affect their engaging in sports activity. The procedures and purpose of the study, including the right to freely withdraw, were explained to the participants, and their informed consent had also been obtained. Each subject agreed to take part in the tests and to practice the Baduanjin exercise for 16 weeks.

During this study period, 110 participants were enrolled and randomly assigned to either the SG group (study group, *n* = 55) or the CG group (control group, *n* = 55). All the 55 participants in SG group received sixteen weeks of Baduanjin training during this study, while other 55 participants in CG group received no training, but maintained their original, daily lifestyle during the same period. There were no differences in anthropometric characteristics between the two groups (*p* > 0.05), as shown in Table 1.

### 2.2. Training Procedures

The 55 subjects in SG group undertook the learning of Baduanjin exercise two weeks prior to the intervention by professional fitness Qigong instructors and were required to complete the program at least 3 times each week, 30–60 minutes each time in the fixed site and in a collective manner, with special personnel recording the attendance and providing guidance. The Baduanjin exercise consists of eight routine movements, with each routine repeated six times in the exercise. The complete exercise can be completed in about ten minutes. There was a five-minute rest between each set during the practice. The 55 subjects in CG group were only asked to maintain their daily activities and to avoid any lower extremity training or practice during the whole study period.

### 2.3. Methods and Tests

A sixteen-channel sEMG system (ME6000 T-16, Mega Electronics Ltd., Kuopio, Finland) was used to record sEMG signals telemetrically during the test. The skin surface was rubbed lightly with sandpaper and cleaned with 75% medical alcohol solution, and excess body hair was shaved when necessary.

After the two-week training for the 55 subjects in SG group, the sEMG test (pretest) was conducted for all the 110 participants. Each participant was tested individually, while following a video presentation and performing the Baduanjin exercise. Test program was conducted by the following steps: (1) three-minute warm-up and stretching; (2) placement of electrodes in the middle of the muscles bellies, with a 10 mm separation of the two active electrodes [13]; (3) recording the sEMG signals by the ME6000 system, while the participant performed Baduanjin exercise. Before and after sixteen weeks of intervention of Baduanjin, the same sEMG tests (posttests) were conducted for all the 110 participants.

### 2.4. Data Analysis

The sEMG signals were collected with a sample of 1000 Hz. MegaWin software was used to compute and analyze the data. In this study, three indexes (RMS, AEMG, and IEMG) were used to evaluate the lower extremity muscle strength and two indexes (MPF and MF) for evaluating muscular fatigue. Statistical analysis was performed using SPSS software (version 19.0, SPSS Inc., Chicago, IL, USA). In the analysis, a two-way ANOVA for repeated measures (2 groups × 2 times) assessed the significance of changes between the pre- and posttests. Tukey's method was also used for post hoc comparison, in cases where ANOVA showed statistically significant differences. Differences between the two groups were considered significant at *p* < 0.05.

## 3. Results

**Figure 1:**
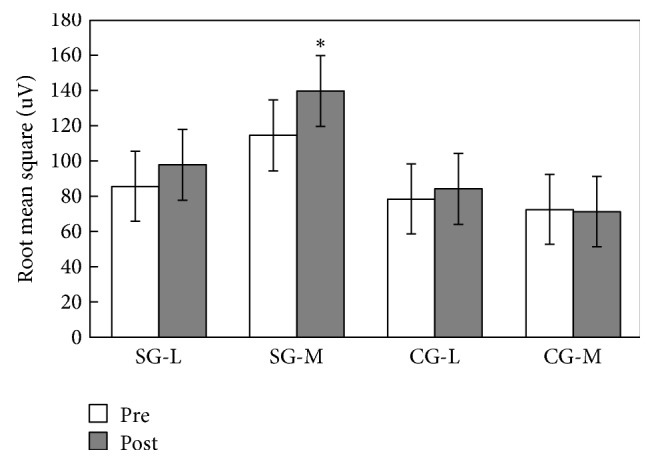
Root mean square in the SG and CG groups (mean ± SD). The *∗* indicates significant difference between the pre- and post-Baduanjin exercise values. SG-L: vastus lateralis of the SG; SG-M: vastus medialis of the SG; CG-L: vastus lateralis of the CG; CG-M: vastus medialis of the CG.

The root mean square (RMS) was not significantly different (in vastus lateralis, *p* = 0.065 > 0.05, *η*^2^ = 0.192; in vastus medialis, *p* = 0.135 > 0.05, *η*^2^ = 0.349) between the study and control groups (SG and CG) before intervention (Table 2). After sixteen weeks of exercise, the simple, main effect comparisons within the SG revealed that there were significant increases of RMS values in vastus medialis (in vastus lateralis, *p* = 0.075 > 0.05, *η*^2^ = 0.677; in vastus medialis, *p* = 0.001 < 0.05, *η*^2^ = 0.60). No significant difference was found in the CG group, neither in vastus lateralis (*p* = 0.125 > 0.05, *η*^2^ = 0.12) nor in the vastus medialis (*p* = 0.829 > 0.05, *η*^2^ = 0.03) (Figure 1).

**Figure 2:**
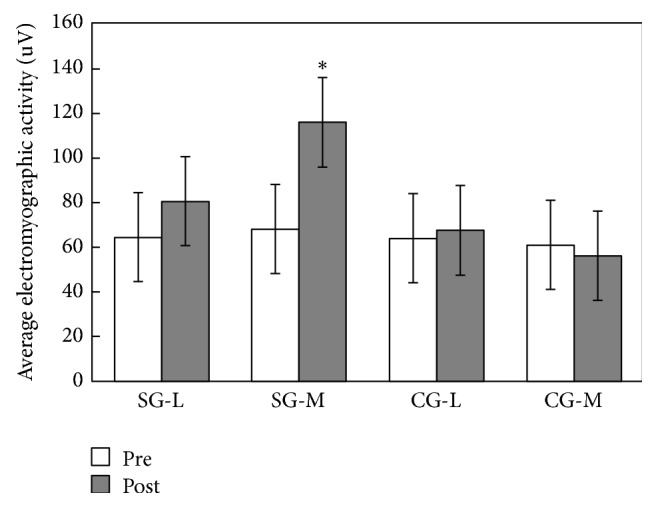
Average electromyographic activity in the SG and CG (mean ± SD). The *∗* indicates significant difference between the pre- and post-Baduanjin exercise values.

The average electromyographic activity (AEMG) was not significantly different (in vastus lateralis, *p* = 0.075 > 0.05, *η*^2^ = 0.162; in vastus medialis, *p* = 0.55 > 0.05, *η*^2^ = 0.25) between study and control groups (SG and CG) before intervention, with the SG showing a significant increase of amplitudes (in vastus lateralis, *p* = 0.085 > 0.05, *η*^2^ = 0.719; in vastus medialis, *p* = 0.001 < 0.05, *η*^2^ = 0.461) after 16 weeks of exercise. No significant difference was found in the CG (in vastus lateralis, *p* = 0.307 > 0.05, *η*^2^ = 0.055; in vastus medialis, *p* = 0.250 > 0.05, *η*^2^ = 0.07) (Figure 2).

**Figure 3:**
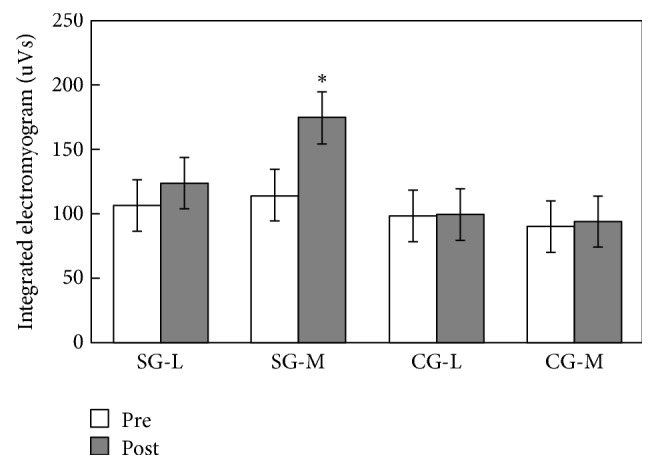
Integrated electromyogram in the SG and CG groups (mean ± SD). The *∗* indicates significant difference between the pre- and post-Baduanjin exercise values.

There was no significant difference between study and control groups (SG and CG) in their integrated electromyogram (IEMG) before intervention (in vastus lateralis, *p* = 0.096 > 0.05, *η*^2^ = 0.352; in vastus medialis, *p* = 0.146 > 0.05, *η*^2^ = 0.497). The SG showed significant increases in the values of IEMG (in vastus lateralis, *p* = 0.065 > 0.05, *η*^2^ = 0.53; in vastus medialis, *p* = 0.015 < 0.05, *η*^2^ = 0.675), after their Baduanjin exercise, while the CG showed no significant change (in vastus lateralis, *p* = 0.779 > 0.05, *η*^2^ = 0.004; in vastus medialis, *p* = 0.380 > 0.05, *η*^2^ = 0.041) (Figure 3).

**Figure 4:**
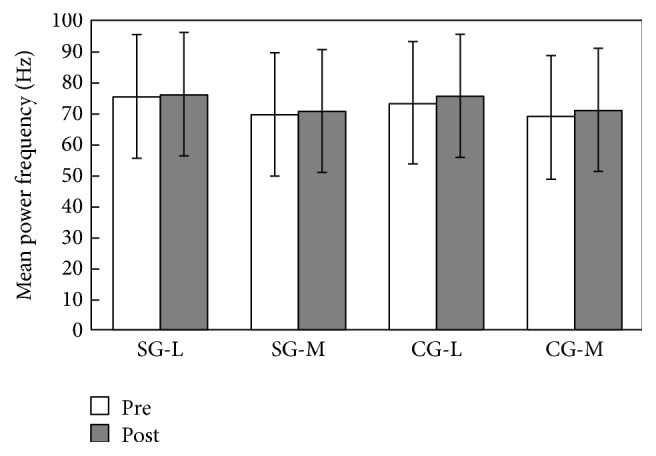
Power frequency in the SG and CG groups (mean ± SD).

Regarding mean power frequency (MPF), no significant differences were found between two groups (in vastus lateralis, *p* = 0.617 > 0.05, *η*^2^ = 0.007; in vastus medialis, *p* = 0.935 > 0.05, *η*^2^ = 0.000). Also no significant difference was found in within-group main effect comparisons for both two groups: for the SG group, in vastus lateralis, *p* = 0.846 > 0.05, *η*^2^ = 0.002; in vastus medialis, *p* = 0.664 > 0.05, *η*^2^ = 0.01; for the CG group, in vastus lateralis, *p* = 0.392 > 0.05, *η*^2^ = 0.039; in vastus medialis, *p* = 0.128 > 0.05, *η*^2^ = 0.118 (Figure 4).

**Figure 5:**
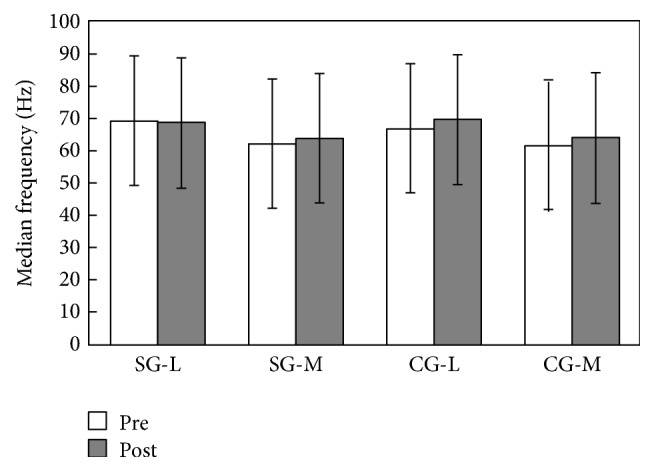
Median frequency in the SG and CG (mean ± SD).

The values of median frequency were not significantly influenced by the sixteen weeks of Baduanjin exercise. The differences between the study and control groups (SG and CG) were not significant (in vastus lateralis, *p* = 0.965 > 0.05, *η*^2^ = 0.000; in vastus medialis, *p* = 0.937 > 0.05, *η*^2^ = 0.000). Further analysis showed no significant difference in within-group, main effect comparisons for both groups: in SG vastus lateralis, *p* = 0.915 > 0.05, *η*^2^ = 0.001; in SG vastus medialis, *p* = 0.440 > 0.05, *η*^2^ = 0.032; in CG vastus lateralis, *p* = 0.224 > 0.05, *η*^2^ = 0.077; in CG vastus medialis, *p* = 0.420 > 0.05, *η*^2^ = 0.2 (Figure 5).

## 4. Discussion

The purpose of this study was to assess the effects of practicing Baduanjin exercises on the lower extremities of subjects using surface electromyography (sEMG) analysis.

EMG signal analysis is widely used as a clinical diagnosis method and for biomedical applications [14]. Parameters in frequency (e.g., MPF) of the EMG were used to analyze muscle fatigue, and parameters in amplitude (e.g., RMS) of EMG can be used to analyze the recruitment of muscle fibers during contraction [15].

Previous published studies indicate that twelve to twenty-four weeks of Qigong-type of exercise (e.g., Baduanjin and Tai Chi Chuan) appears to have positive effect on the muscular strength of the lower extremities [6, 16]. In this study, muscle strength was significantly increased in the SG after 16 weeks of Baduanjin exercise, expressed as root mean square (RMS), integrated electromyogram (IEMG), and average electromyographic activity (AEMG). In the time domain, the lager amplitude corresponds to more recruitment of muscle fibers [15]. The values of RMS, IEMG, and AEMG in the SG were increased, but only significant in vastus medialis. This indicates that Baduanjin exercise enhances the physical function of lower extremities, while the CG showed no significant improvement. The values of RMS, IEMG, and AEMG show significant change only in vastus medialis; one possible reason is that Baduanjin exercise significantly enhanced the muscle strength of the vastus medialis [6]. Another reason is that the intervention period in this study was only 16 weeks, which is too short to improve the muscle strength of vastus lateralis.

One research [4] reported that 11 female subjects with knee OA, who performed Baduanjin five times a week for 8 weeks, had significantly reduced pain, stiffness, and disability, as well as improved strength of their quadriceps and their aerobic ability (with no adverse effects reported). Another study reported that 12 subjects, who performed the 108-form Tai Chi Chuan, three times per week, for 12 weeks, had significantly increased muscular strength of their knee extensors [17]. Moreover, in a recent study, muscular strength of the knee was measured by isokinetic testing at 30°/s. The study group practicing Tai Chi Chuan demonstrated greater eccentric muscular strength in both knee extensors and flexors than the control group [18]. In the Baduanjin exercise, separate movements and postures focus on different parts of the body and, of these, there are three that especially strengthen the muscles of the legs. The degree of knee flexion required to perform the single-leg stance of Tai Chi Chuan is considered to be a key element for improving leg muscle strength [19].

Regarding mean power frequency (MPF) and median frequency (MF), no significant increases were noted among the SG participants, who received sixteen weeks of Baduanjin training. And neither were there significant differences between the SG and CG. Regarding frequency domain, the values of MPF and MF are sensitively related to muscular fatigue [15]. The results in the analysis indicated that no significant fatigue resulted from Baduanjin training. As also indicated in previous studies, Baduanjin exercise could be an appropriate, low-intensity exercise for both healthy adults and people with OA. Positive effects were also reported when Baduanjin exercise was practiced as part of OA treatment [4].

This study was initiated to explore the effects of Baduanjin training, using EMG analysis as the method for assessing lower extremities activity. Being a pilot study, small sample sizes were used. Qigong and Tai Chi exercises are considered to be operationally equivalent for the promotion of health and wellness [6]. And larger sample sizes should be used in order to apply the results to the overall population. Other effects of Baduanjin training should be evaluated in further studies.

## 5. Conclusions

This study indicates that performing sixteen weeks of Baduanjin training can improve the physical strength and function of the lower extremities of healthy adults, with no adverse effects reported from the exercise during the training period. The low intensity of this type of exercise was also established by this study. And, therefore, Baduanjin may be considered as a suitable, no-risk exercise for healthy adults.

## Figures and Tables

**Table 1:** Means ± SD of physical characteristics in the SG and CG groups.

	SG (*N* = 55)	CG (*N* = 55)
Age (yrs)	35.5 ± 16.0	32.9 ± 13.0
Height (m)	1.66 ± 0.08	1.64 ± 0.07
Body mass (kg)	63.5 ± 11.2	61.4 ± 11.8

**Table 2:** Root mean square (RMS), average electromyographic activity (AEMG), integrated electromyogram (IEMG), mean power frequency (MPF), and median frequency (MF) before and after sixteen weeks, for the SG and CG. Data are means ± SD.

		Study group (*N* = 55)		Control group (*N* = 55)	
		Pre	Post	Pre	Post
RMS (uV)	Vastus lateralis	85.85 ± 41.70	98.00 ± 39.33	78.50 ± 31.15	84.30 ± 29.14
	Vastus medialis	80.05 ± 33.74	139.90 ± 44.98^*∗*^	72.35 ± 23.60	71.45 ± 23.67
AEMG (uV)	Vastus lateralis	64.35 ± 30.57	80.55 ± 32.27	63.85 ± 27.33	67.25 ± 26.94
	Vastus medialis	67.75 ± 30.36	115.80 ± 58.00^*∗*^	60.70 ± 20.53	55.85 ± 20.86
IEMG (uVs)	Vastus lateralis	106.13 ± 59.74	123.73 ± 53.57	98.28 ± 48.66	99.38 ± 40.55
	Vastus medialis	114.45 ± 41.81	174.43 ± 66.44^*∗*^	90.1 ± 41.55	93.78 ± 52.16
MPF (Hz)	Vastus lateralis	75.55 ± 9.05	76.25 ± 12.58	73.45 ± 7.85	75.55 ± 8.39
	Vastus medialis	69.75 ± 11.28	70.75 ± 11.18	69.00 ± 8.65	71.15 ± 6.81
MF (Hz)	Vastus lateralis	69.02 ± 10.55	68.62 ± 11.32	66.71 ± 11.42	69.66 ± 11.94
	Vastus medialis	62.08 ± 6.99	63.68 ± 7.55	61.74 ± 7.66	63.86 ± 6.79

^*∗*^Value after sixteen weeks of Baduanjin exercise is significantly larger than the value before training (*p* < 0.025).
